# Supplementary material for: Molecular Modelling Study and Antibacterial Evaluation of Diphenylmethane Derivatives as Potential FabI Inhibitors
Source: Molecules. 2023 Mar 28;28(7):3000. doi: 10.3390/molecules28073000 (PMC10095751; doi:10.3390/molecules28073000)
Supplement: Supplementary file 1 [file molecules-28-03000-s001.zip › molecules-2251475-supplementary.pdf]

Molecular Modelling study and antibacterial evaluation of diphenylmethane derivatives as potential FabI inhibitors

Shaima Hasan [1], Kawthar Kayed [1,2], Rose Ghemrawi [1,2], Nezar Al Bataineh [1,2], Radwa E. Mahgoub[2], Rola Audeh [1], Raghad Aldulaymi [2], Noor Atatreh [1,2], Mohammad A. Ghattas [1,2,*]

1 College of Pharmacy, Al Ain University, Abu Dhabi 64141, United Arab Emirates
2 AAU Health and Biomedical Research Center, Al Ain University, Abu Dhabi 64141, United Arab Emirates
* Correspondence: mohammad.ghattas@aau.ac.ae; Tel.: +971-26133275

Table S1. ADME analysis for MN02 derivatives by SWISSADME software.

		Compound ID									
		RK01	RK02	RK03	RK04	RK05	RK06	RK07	RK08	RK09	RK10
Pharmacokinetics	GI absorption	High	High	High	High	High	High	High	High	High	High
	BBB permeant	Yes	Yes	Yes	Yes	Yes	Yes	Yes	Yes	Yes	Yes
	P-gp substrate	No	No	No	No	No	No	No	No	No	No
	CYP1A2 inhibitor	Yes	Yes	Yes	Yes	Yes	Yes	Yes	Yes	Yes	Yes
	CYP2C19 inhibitor	**No**	Yes	Yes	Yes	Yes	Yes	Yes	Yes	Yes	Yes
	CYP2C9 inhibitor	Yes	Yes	Yes	Yes	Yes	Yes	**No**	Yes	Yes	Yes
	CYP2D6 inhibitor	**No**	**No**	**No**	Yes	**No**	**No**	**No**	**No**	Yes	**No**
	CYP3A4 inhibitor	**No**	**No**	**No**	**No**	**No**	**No**	**No**	Yes	**No**	**No**
	Bioavailability score	0.55	0.55	0.55	0.55	0.55	0.55	0.55	0.55	0.55	0.55
Druglike	Lipinski	Yes	Yes	Yes	Yes	Yes	Yes	Yes	Yes	Yes	Yes
	Ghose	Yes	Yes	Yes	Yes	Yes	Yes	Yes	Yes	Yes	Yes

	Veber	Yes	Yes	Yes	Yes	Yes	Yes	Yes	Yes	Yes	Yes
	Egan	Yes	Yes	Yes	Yes	Yes	Yes	Yes	Yes	Yes	Yes
	Muegge	Yes	Yes	**No**	**No**	Yes	Yes	Yes	Yes	Yes	Yes
**Medicinal Chemistry**	PAINS	0 alert	0 alert	0 alert	0 alert	0 alert	0 alert	0 alert	0 alert	0 alert	0 alert
	Brenk	0 alert	0 alert	1 alert Polycyclic_aromatic_hydrocarbon _3	0 alert	1 alert Michael acceptor	0 alert	0 alert	0 alert	0 alert	0 alert
	Leadlikeness	LopP >3.5	LopP >3.5	LopP >3.5	LopP > 3.5	LopP >3.5	LopP >3.5	LopP >3.5	LopP >3.5	LopP >3.5	LopP >3.5
